# Assessment of oral health related quality of life in children and adolescents affected by traumatic dental injuries: a hospital-based study

**DOI:** 10.1007/s40368-026-01243-6

**Published:** 2026-06-22

**Authors:** D. Pamei, N. Tewari, P. Haldar, V. Mathur, K. Bansal, M. Rahul, M. Monica, A. K. Lokade

**Affiliations:** 1 Pediatric and Preventive Dentistry, Centre for Dental Education and Research, All India Institute of Medical Sciences, New Delhi, India; 2 Department of Dental Medicine, Karolinska Institutet, Stockholm, Sweden; 3 Centre for Community Medicine, All India Institute of Medical Sciences, New Delhi, India; 4 Department of Pediatric and Preventive Dentistry, ESIC Hospital, Delhi, India

**Keywords:** Tooth injuries, Quality of life, Child, Adolescent, Pediatric dentistry

## Abstract

**Background:**

Traumatic dental injuries (TDI) represent a significant public health concern, affecting nearly one in five children worldwide. Beyond clinical sequelae, TDI can profoundly influence functional ability, emotional well-being, and social interactions. However, evidence from hospital-based settings, particularly across diverse regions, remains limited.

**Objective:**

This study aimed to assess the oral health-related quality of life (OHRQoL) of children and adolescents presenting with TDI to a tertiary care hospital and to identify demographic, clinical, and trauma-related factors associated with severe impairment.

**Methods:**

A cross-sectional analytical study was conducted among 266 children aged 8–16 years who had experienced TDI within the preceding 12 months. Dental injuries were diagnosed according to the International Association of Dental Traumatology (IADT, 2020) guidelines. OHRQoL was evaluated using the Child Oral Impacts on Daily Performance (C-OIDP) questionnaire. Associations between OHRQoL and demographic, clinical, and trauma-related variables were examined using univariate and multivariable logistic regression analyses.

**Results:**

Maxillary central incisors were most frequently affected, with uncomplicated crown fractures being the most common injury type (64.6%). Severe injuries accounted for about 32% of cases, and soft-tissue involvement was observed in 56% of patients. Most patients experienced delayed emergency care, with only 14% seeking treatment within 24 hours of injury. OHRQoL was substantially compromised: 93% reported awareness of broken teeth, 78% experienced sensitivity, and 67% expressed aesthetic concerns. Functional impacts included difficulties in eating (63%) and cleaning the mouth (48%), while emotional and social impacts included reluctance to smile (52%) and reduced interaction with peers (51%). Severe OHRQoL impairment (OIDP ≥ 8) was observed in about 26% of participants. Multivariable analysis identified trauma severity, repeated episodes, soft-tissue injury, aesthetic concerns, and eating difficulties as associated factors of severe OHRQoL impairment.

**Conclusion:**

The severe impact on the OHRQoL was seen in about 26% participants. Greater injury severity, soft-tissue involvement, and repeated trauma episodes were associated with poorer oral health-related quality of life.

**Supplementary Information:**

The online version contains supplementary material available at https://doi.org/10.1007/s40368-026-01243-6.

## Introduction

Traumatic dental injuries (TDI) have emerged as a major public health concern, with a global prevalence ranging from 15.2 to 22.7% across different age groups (Petti et al. 2018). Each traumatic episode presents unique clinical challenges, often involving anxious children and distressed parents, which can complicate diagnosis, management, and follow-up (Tewari et al. 2019). Children may experience pain, bleeding, mobility, disfigurement, or fractured and missing teeth as immediate consequences of TDI. Inadequate or delayed management can further result in complications such as discoloration, swelling, sinus tract formation, and resorptive lesions affecting the root and periodontal tissues. Trauma to the primary dentition may also adversely influence the development of permanent successor teeth (Al-Nazhan et al. 1995; Khan et al. 2019). Beyond these clinical sequelae, the psychological and social consequences of dental injuries in children and adolescents remain insufficiently recognized (Traebert et al. 2018; Rodd and Nobel. 2019).

Oral Health-Related Quality of Life (OHRQoL) provides a comprehensive framework for evaluating the functional, emotional, and social consequences of oral conditions. It is a multidimensional construct that encompasses self-perception, expectations, satisfaction with care, and functional as well as emotional well-being. Recognizing its significance, the World Health Organization incorporated OHRQoL into the Global Oral Health Program (Sischo and Broder. 2011; Gilchrist et al. 2014). Several validated instruments are available for assessing OHRQoL in children, including the Early Childhood Oral Health Impact Scale (ECOHIS), the Child Oral Impacts on Daily Performance (C-OIDP), the Scale of Oral Health Outcomes for 5 years old children (SOHO-5), and the Child Perception Questionnaires (CPQ 8–10 and CPQ 11–14) (Gilchrist et al. 2014). These tools assess the extent to which specific oral conditions affect daily functioning across the age-appropriate physical, emotional, and social domains (Gilchrist et al. 2014; Abanto et al. 2014, 2015).

Although oral health is influenced by multiple biological, social, and environmental factors, understanding the specific impact of TDI on OHRQoL is essential (Tewari et al. 2023). Despite systematic reviews demonstrating a clear association between dental trauma and impaired quality of life, the existing literature is predominantly derived from studies conducted in Latin America, with limited evidence from other regions (Tewari et al. 2023; Lopez et al. 2019; Borges et al. 2017; Zaror et al. 2018; Antunes et al. 2020; Das et al. 2022). Furthermore, many studies exhibit methodological limitations, such as retrospective injury assessment, absence of standardized classification criteria, or reliance on school-based study designs. Assessment of TDi in school settings may limit interpretation due to recall bias and other subjective confounding factors. To date, only a limited number of studies have been conducted in clinical settings, and they have yielded inconsistent findings regarding the effect of TDI on OHRQoL (Giannetti et al. 2007; Berger et al. 2009; Porritt et al. 2011; Bouchardet et al 2014; Magno et al. 2019; Celikel et al 2024). In addition, critical factors such as injury severity, time to presentation, and treatment delays have rarely been examined in relation to OHRQoL (Tewari et al. 2023).

Given these gaps, there is a pressing need for hospital-based evidence that captures the clinical complexity of TDI and their multidimensional impacts on children and adolescents. Therefore, the present study aimed to characterize OHRQoL patterns among children and adolescents with TDI presenting to a tertiary care hospital and to identify demographic, clinical, and trauma-related factors associated with severe impairment in daily functioning.

## Methods

### Study setting and ethical approval

This cross-sectional analytical study was designed in accordance with the Strengthening the Reporting of Observational studies in Epidemiology (STROBE) (Vandenbroucke et al. 2007) and the OHStat Guidelines (Best et al. 2024). The study was conducted in the Department of Paediatric Dentistry in a tertiary care hospital. Ethical approval was obtained from the Institutional Ethics Committee (Ref. No. IECPG-643/28.10.2021, OT-01/26.05.2022).

### Study design and sample size

The sample size was calculated based on an expected prevalence of 30% for impaired OHRQoL among children with TDI, as reported by Ramos-Jorge et al. (Ramos-Jorge et al. 2014). Assuming a relative precision of 20% and an anticipated attrition rate of 10%, the sample size was determined to be 250 children.

### Participant recruitment

Children aged 8–16 years presenting to the outpatient clinic with a chief complaint of injury to the tooth/teeth were evaluated by an experienced paediatric dentist. Screening included structured history taking, detailed clinical examination, and appropriate radiographic assessment. Children with a confirmed history of TDI within the preceding 12 months were enrolled consecutively from 20th December 2021 to 19th December 2024.

### Inclusion criteria

- Children aged 8–16 years
- History of any type of TDI within the preceding 12 months
- Written informed parental consent and child assent

### Exclusion criteria

- Prior treatment for the previous dental injury
- Presence of associated maxillofacial injuries
- History of systemic or neurological disorders

### Consent and assent procedures

Information sheets were provided to parents and children in English and the regional language. The purpose of study, study procedures, and participants’ rights were explained in age-appropriate and comprehensible language. Written informed consent was obtained from parents or legally authorized representatives, and assent was obtained from participating children prior to enrolment. 

### Clinical examination and recording

All clinical examinations were performed by a single calibrated examiner. A detailed trauma history was recorded for each patient, including the mechanism and location of injury, time elapsed since the traumatic event, and details of any emergency care received. Dental injuries were classified according to the NA0D classification system (Petti et al. 2022) and were further grouped as “less severe injuries” (Uncomplicated crown fractures, concussion, subluxation) and “more severe injuries” (Complicated crown fracture, crown-root fracture, root fracture, dentoalveolar fracture, and luxation). This severity categorization was based on clinical experience and supported by the categorization used in previous literature (Vieira et al. 2023). Diagnosis and treatment planning were carried out in accordance with the guidelines of the International Association of Dental Traumatology (IADT), and standardized extraoral and intraoral photographs were obtained for documentation and record-keeping purposes (Levin et al. 2020; Bourguignon et al. 2020; Fouad et al. 2020). Emergency care was provided at the first visit when clinically needed. Additional assessments included evaluation of dental caries status using the International Caries Detection and Assessment System II (ICDAS-II) (Ismail et al. 2007) and orthodontic parameters, including facial profile, lip competence, overjet, overbite, and molar relationship (Proffit et al. 2019). For analytical purposes, patients were grouped as caries free or having one or more carious teeth.

### Assessment of oral health-related quality of life

OHRQoL was assessed using the Child Oral Impacts on Daily Performance (C-OIDP) questionnaire (Gherunpong et al 2004; Mathur et al. 2022). This validated instrument measures the impact of oral conditions on eight domains of daily performance and includes a symptom-screening component. Authorized versions of the questionnaire in English and the regional language were available and used in the present study with permission from the developers (Mathur et al. 2022; Basavaraj et al. , 2014). To; ; minimize the acute-phase bias and ethical concerns of prioritizing the treatment of severe injuries, children with less severe injuries or those presenting beyond the acute phase (within 2 weeks after injury) completed the C-OIDP questionnaire at the first visit. In contrast, children with more severe injuries completed the questionnaire at a follow-up visit, seven days after the initial management.

For analytical purposes, the total C‑OIDP score was dichotomized to differentiate patients with mild to moderate impacts from those experiencing severe impairment in OHRQoL from those experiencing severe impairment in the OHRQoL. A cut‑off value of C‑OIDP ≥ 8 was used to define severe impact. The conceptual basis for this dichotomization is supported by the original OIDP framework, which was designed to capture the ultimate consequences of oral disease, namely disability and handicap, rather than early clinical symptoms alone. Accordingly, higher summary scores represent clinically meaningful impairment, justifying the classification of elevated score ranges as severe (Adulyanon and Sheiham. 1996).

In addition, validation studies of child and adolescent versions of the OIDP have consistently demonstrated that higher OIDP scores are associated with poorer self-perceived oral health, greater treatment needs, and more extensive disruption of daily activities, thereby supporting the construct validity of defining higher score thresholds as indicative of severe impact (Gülcan et al. 2014; Amilani et al. 2020).

### Data management and statistical analysis

Data were entered into Microsoft Excel (Microsoft, Redmond, USA) and checked for completeness and accuracy prior to analysis. Statistical analysis was performed using STATA version 16 (StataCorp, USA). Descriptive statistics were generated for all study variables. Since the primary outcome of interest was severe impairment in OHRQoL (dichotomized), inferential analyses focused on binary logistic regression. Univariate and multivariable logistic regression models were used to evaluate associations between severe OHRQoL impairment and trauma characteristics, including injury severity, soft-tissue involvement, frequency of trauma, dental caries status, orthodontic parameters, and delay in reporting for care. A *p* value < 0.05 was considered statistically significant.

Categorical variables were entered into the models as dummy variables with appropriate reference categories. Ordinal variables were treated as ordinal associated factors after confirming a monotonic relationship with the log odds of the outcome. Model building was guided primarily by clinical plausibility and existing literature, supplemented by univariate screening to avoid omission of potentially important associated factors. Variables were not excluded solely on the basis of non-significant univariate *p* values. There was no missing data for variables included in the multivariable analysis. Model adequacy was considered in relation to the number of severe outcome events observed. The final multivariable model adhered to the recommended events per variable criterion, with approximately 10 or more severe outcome events per predictor variable, thereby reducing the risk of overfitting. Accordingly, the number of associated factors included in the final model was restricted to ensure model stability.

## Results

### Study population

A total of 266 children and adolescents aged 8–16 years were included in the study. The largest age group was 8–10 years (42%), followed by 11–13 years (40%) and 14–16 years (18%). Males comprised 61% of the study population and nearly half of the parents (47%) had education up to high school (Table 1).

**Table 1:** Details of the patients’ characteristics- age, gender, education level of the patient, and educational qualifications of the parents

Characteristics	Frequency		Percentage
Age in years			
8–10	112	42%	
11–13	106	40%	
14–16	48	18%	
Gender			
Males	163	61%	
Females	103	39%	
Educational qualification of the parent			
≤ High School	125	47%	
Higher Secondary School	36	14%	
≥ Graduate	105	39%	

### Characteristics of traumatic dental injuries

TDI predominantly affected the maxillary central incisors, with the right central incisor involved in approximately 48% and the left central incisor in 44% of the patients. The majority of patients (96%) experienced a single traumatic episode. Less severe injuries accounted for approximately 68% of patients, with uncomplicated crown fractures (≈65%) being the most common injury type. More severe injuries were observed in about 32% patients, with complicated crown fractures being the most frequent within this group (≈29%). Soft-tissue injuries were present in 56% of the patients with lacerations (44%) and contusions (42%) as the most common injury types. The leading causes of trauma were being struck while playing (46%) and falls (38%) (Table 2).

**Table 2:** Details of the characteristics of traumatic dental injuries

Characteristics	Frequency	Percentage
Tooth notation		
Right maxillary central incisor (11)	184	48.2%
Left maxillary central incisor (21)	167	43.7%
Left maxillary lateral incisor (22)	11	2.9%
Right maxillary central incisor (12)	10	2.6%
Left mandibular lateral incisor (32)	4	1.1%
Left mandibular central incisor (31)	3	0.8%
Right mandibular central incisor (41)	2	0.5%
Right mandibular lateral incisor (42)	1	0.2%
Frequency of trauma		
Once	256	96%
Twice	10	4%
Type of trauma		
No sign of injury	0	0%
Less severe injuries	181	68.1%
Uncomplicated crown fracture (enamel infraction, enamel fracture, enamel dentin fracture)	172	64.6%
Concussion, subluxation	9	3.4%
More severe injuries	85	31.9%
Complicated crown fracture	77	28.9%
Intrusive, extrusive, lateral luxation	3	1.1%
Avulsion	5	1.9%
Associated with soft tissue injury		
No	117	44%
Yes	149	56%
Type of soft tissue injury		
Contusion	111	42%
Abrasion	38	14%
Laceration	117	44%
Description of injury		
Fall from stairs/height	49	18%
Fall/ slip while walking	53	20%
Hit while playing	123	46%
Fall from bicycle	28	11%
Road traffic accident	12	5%

### Emergency care and treatment-seeking behavior

Emergency treatment was not received in 71% of patients. Only 27% of patients consulted a dentist immediately following the traumatic event. Delays in reporting for definitive care were substantial: 44% of patients presented after 1–3 months, and 16% reported for care after 7–12 months. Only 14% of patients sought care within the first 24 hours following trauma (Table 3).

**Table 3:** Details of emergency treatment, whether dentist was consulted, and time elapsed till patient reported for the comprehensive care

When Emergency treatment was provided	Frequency	Percentage
Not provided	188	70.7%
30 minutes	56	21.1%
1 hour	4	1.5%
1 day	18	6.7%
Dentist consulted		
Yes	71	27%
No	195	73%
Time elapsed till patient reported for the comprehensive care		
≤1day	38	14%
2–7 days	41	15%
8–30 days	7	2.6%
1–3 months	116	44%
4–6 months	22	8.4%
7–12 months	42	16%

### Orthodontic characteristics and dental caries

A convex facial profile was observed in 51% of patients, while lip incompetence was noted in 28%. Increased overjet (> 5.5 mm) was present in 26%, and deep bite (≥75%) in 16% of patients. The majority of patients (76%) exhibited a Class I molar relationship. Dental caries was common, with 32% having at least one carious tooth; approximately 10% had four affected teeth (Table 4).

**Table 4:** Details of orthodontic parameters and number of teeth with dental caries

Parameters	Frequency	Percentage
Orthodontic parameters		
Facial profile		
Straight	130	49%
Convex	136	51%
Concave	0	0%
Lip Competency		
Competent	191	72%
Incompetent	75	28%
Overjet		
1–4 mm	131	49%
4.5–5 mm	68	26%
5.5–7 mm	39	15%
>7 mm	28	11%
Overbite		
25%	131	49.2%
50%	92	34.6%
75%	31	11.7%
100%	12	4.5%
Molar relation		
Class 1	201	76%
Class 2	65	24%
Number of caries affected teeth		
0	181	68%
1-3	48	18 %
4-6	36	13.6%
>6	1	0.4%

### Oral impacts on daily performance (OIDP)

OHRQoL was markedly affected among the study participants. The most frequently reported symptoms were awareness of decay or a broken tooth (93%), toothache or sensitivity (78%), and aesthetic concerns related to tooth color, shape, size, or position (67%).

Functional impacts were also common, with difficulties in eating reported by 63% of patients, cleaning the mouth by 48%, and relaxing or sleeping by 27%. Emotional and social impacts included difficulties with feelings (39%), reluctance to smile or laugh (52%), and reduced interaction with peers (51%). The total OIDP severity score ranged from 0 to 18 with a mean score of 5.1 and a median score of 4. Based on the predefined cut‑off value, approximately 26% of patients experienced severe impacts on daily performance (OIDP score ≥8) indicating severe impairment of OHRQoL (Table 5).

**Table 5:** Responses of patients for different domains of the oral impact of daily performance scale

Domains of OIDP		Frequency	Percentage
Had toothache or sensitive teeth			
No		59	22%
Yes		207	78 %
Had bleeding or swollen gums			
No		136	51%
Yes		130	49%
Been aware of decay in your teeth or a broken adult tooth			
No		18	7%
Yes		248	93%
Had ulcers or a loose baby tooth			
No		223	84%
Yes		43	16%
Had a problem because of tooth colour, shape, size or position			
No		88	33%
Yes		178	67%
Difficulties in eating			
0		98	37%
1		103	39%
2		46	17%
3		19	7%
Difficulties in speaking			
0		214	80%
1		47	18%
2		4	1.6%
3		1	0.4%
Difficulties in cleaning mouth			
0		138	52%
1		72	27%
2		34	13%
3		22	8%
Difficulties in relaxing (including sleeping)			
0		193	73%
1		30	11%
2		20	7.5%
3		23	8.5%
Difficulties with feelings (more impatient, irritable, easily upset)			
0		163	61%
1		55	21%
2		23	8.6%
3		25	9.4%
Difficulties in smiling or laughing			
0		127	48%
1		66	25%
2		38	14%
3		35	13%
Difficulties in doing schoolwork			
0		221	83%
1		34	13%
2		5	1.9%
3		6	2.1%
Difficulty in mixing with friends or other people			
0		130	48.9%
1		66	24.8%
2		44	16.5%
3		26	9.8%
Overall		Mean	Median
Total Severity	Min=0, Max= 18	5.1	4
OIDP score ≤7	Less severe	197	74.1%
OIDP score ≥8	Severe	69	25.9%

### Univariate logistic regression

**Fig. 1:**
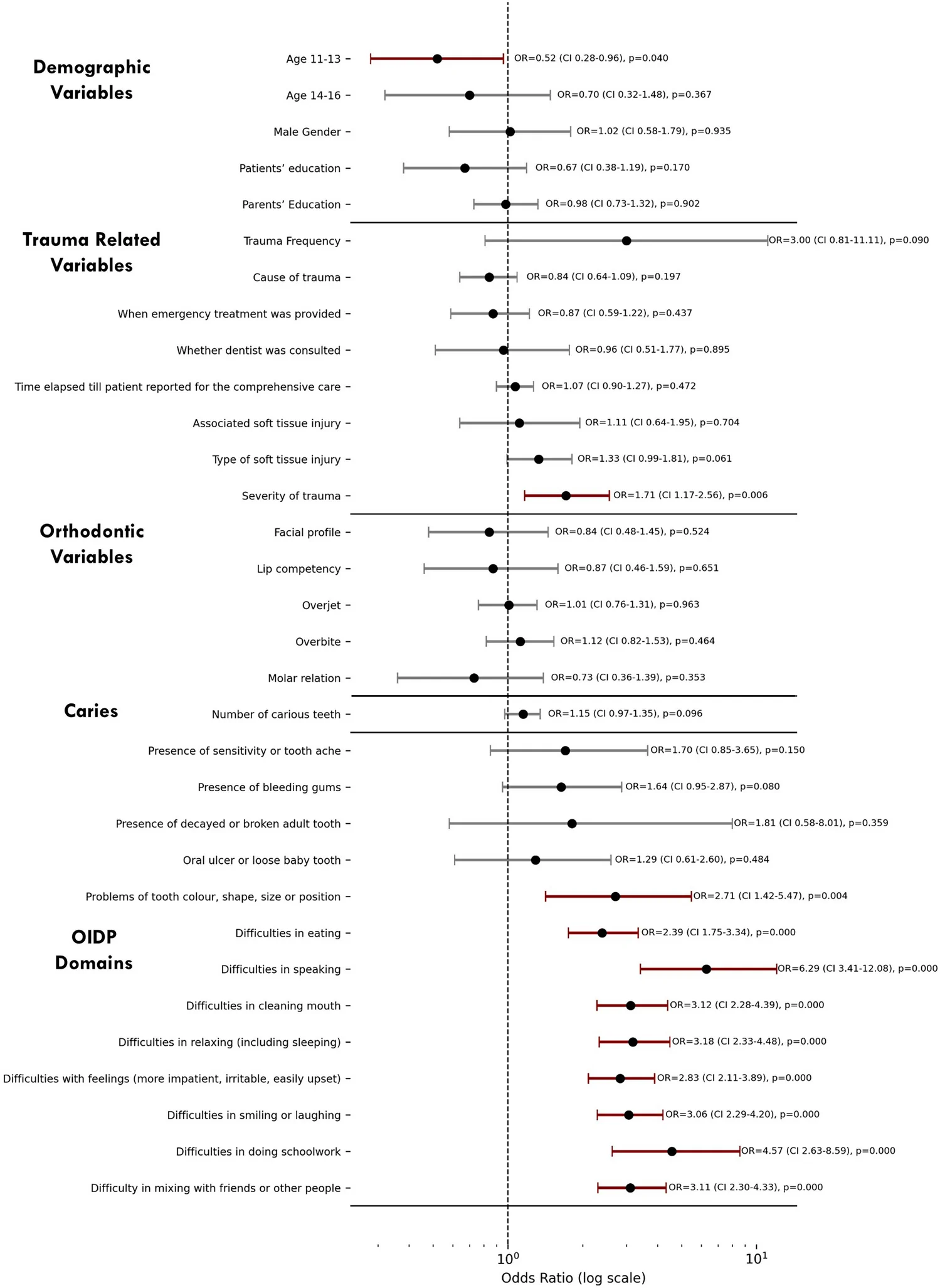
Forest plot showing the univariate binary logistic regression analysis with dependent variable as total score of OIDP ≥8 (severe) versus the total OIDP score ≤7 (less severe); sub grouped into demographic variables, trauma-related variables, orthodontic variables, and dental caries

Several variables were significantly associated with severe OIDP (≥ 8). The severity of trauma was strongly associated with severe OIDP (OR 1.7, 95% CI 1.2–2.6; *p*=0.006). Among demographic factors, children aged 11–13 years had significantly lower odds of severe OIDP (OR 0.5, 95% CI 0.3–0.9; *p* = 0.040). The gender of the patient, and parental education were not significantly associated with severe OIDP. None of the orthodontic parameters or the number of carious teeth were significantly associated with severe OIDP (Fig. 1, Supplementary File 1).

### Multivariable logistic regression

**Fig. 2:**
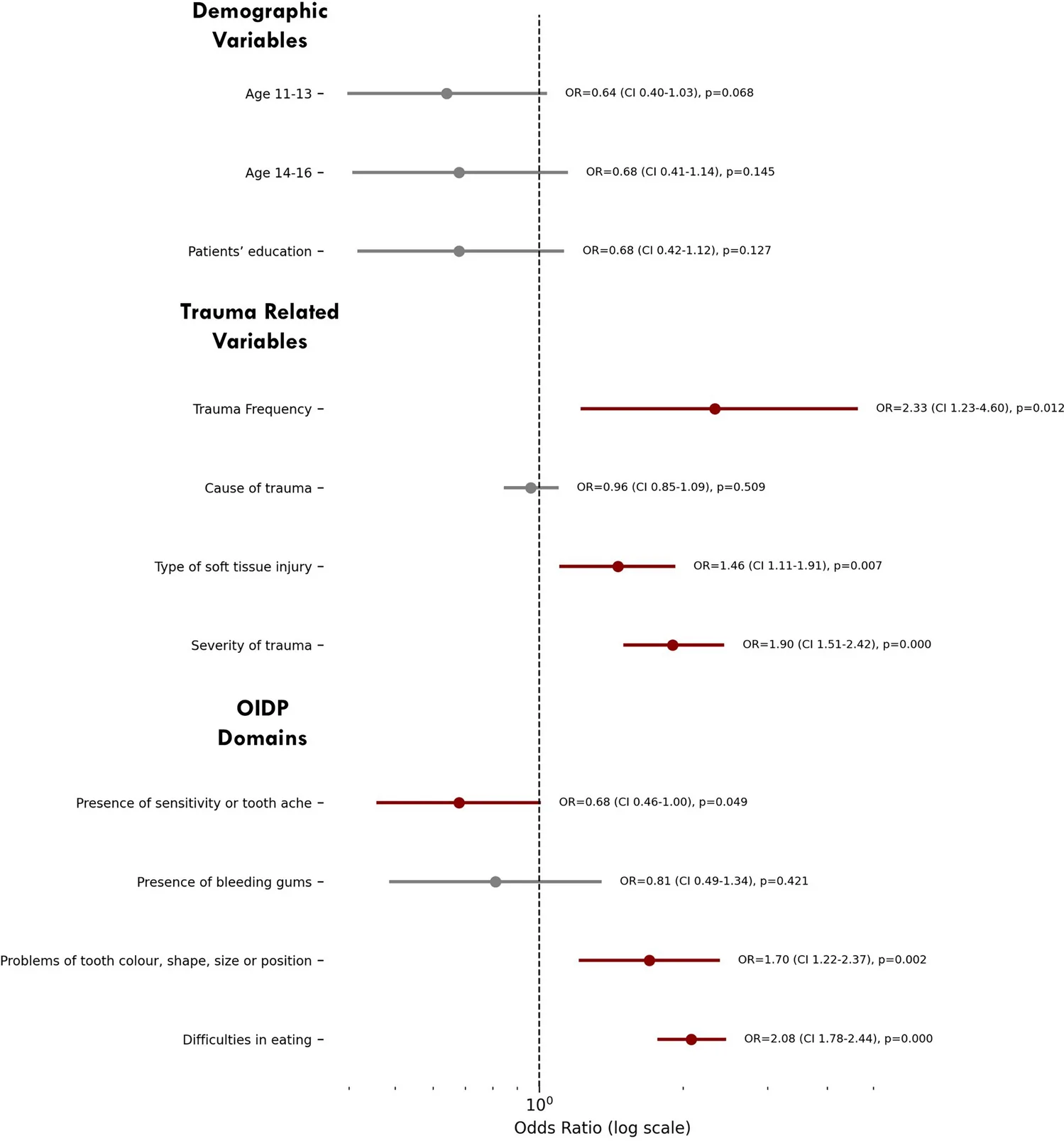
Forest plot showing the multivariable binary logistic regression analysis with the dependent variable as total score of OIDP ≥8 (severe) versus the total OIDP score ≤7 (less severe); sub grouped into demographic variables, and trauma-related variables

After adjustment, several variables remained independently associated with severe OIDP. They included trauma frequency (OR 2.3, 95% CI 1.2–4.6; *p* = 0.012), type of soft-tissue injury (OR 1.5, 95% CI 1.1–1.9; *p* = 0.007), and severity of trauma (OR 1.9, 95% CI 1.5–2.4; *p*<0.001). Age, and cause of trauma did not retain significance in the adjusted model (Fig. 2, Supplementary File 2).

## Discussion

This hospital-based study investigated the impact of traumatic dental injuries on the oral health-related quality of life of children and adolescents aged 8–16 years. The findings demonstrate that TDI impairs daily functioning, emotional well-being, and social interactions, with around one-quarter of participants experiencing severe impact on OHRQoL. These results reinforce the multidimensional burden of dental trauma and contribute valuable evidence from a region where high-quality data remains limited, an important gap highlighted in recent systematic and umbrella reviews (Borges et al. 2017; Zaror et al. 2018; Lopez et al. 2019; Antunes et al. 2020; Tewari et al. 2023).

The demographic profile of affected children in the present study marked by a mean age 10.8 years and a predominance of males, was consistent with epidemiological patterns reported globally. Studies from India, Brazil, Europe, and the Middle East consistently demonstrated a higher prevalence of TDI among boys, commonly attributed to greater participation in sports and risk-prone activities (Petti et al. 2018; Glendor 2008). Similarly, an umbrella review conducted in 2023 identified male predominance across global datasets and highlighted the persistent lack of robust data from under-represented regions such as South Asia and Africa (Tewari et al. 2023).

The distribution of injury types in the present study closely aligns with international pattern reported in the literature (Dame-Teixeira et al. 2013). Enamel-dentin fractures and complicated crown fractures were the most frequently recorded injuries, consistent with findings from studies conducted in Germany, the Czech Republic, Turkey, and Brazil. The prevalence of avulsion (9.4%) was comparable to reports from hospital-based studies in Europe and the Middle East, but higher than the school-based surveys. This difference likely reflects the tertiary care setting of the present study, where more severe cases are referred and managed (Petti et al. 2018; Glendor 2008; Dame-Teixeira et al. 2013; Rocha and Cardoso. 2001; Bücher et al. 2013; Hecova et al. 2010; Atabek et al. 2014).

A key observation was the substantial delay in seeking care, with a median reporting time of two weeks and only 37% receiving any form of emergency management. Similar delays have been reported by Chaudhary et al and Al‑Nazhan et al, who noted that aesthetic concerns and pain, rather than awareness of urgency often drive care-seeking behavior following TDI (al-Nazhan et al. 1995; Chaudhary et al. 2021). The low rate of appropriate emergency care may be attributed to inadequate parental knowledge and limited awareness among teachers, sports coaches, and medical professionals regarding emergency management of TDI, as highlighted in several systematic reviews (Tewari et al. 2020, 2021a, 2021b, 2021c, 2023b). Collectively, these findings underscore the urgent need for targeted public health education and training initiatives aimed at early recognition and adequate emergency care.

The present study demonstrated that children with more severe TDI experienced greater impairment across multiple C-OIDP domains, particularly difficulties related to eating, cleaning the mouth, relaxing, and interacting with peers. Injuries involving pulp exposure and damage to the supporting structures of the teeth are commonly associated with pronounced symptoms such as pain, sensitivity, and bleeding, and if inadequately managed, may lead to adverse long-term complications (Tewari et al. 2019). These findings are consistent with observations reported by Porritt et al. and Magno et al., who similarly documented significant negative effects of TDI on functional, emotional, and social well-being (Porritt et al. 2011; Magno et al. 2019). However, it may also be important to understand the specific concerns such as aesthetics, function, and psychological effects that are more closely associated with risk of greater effect on OHRQoL. This may add clarity to the present understanding that the acute and visible consequences of trauma may overshadow chronic oral conditions in shaping children’s perceived quality of life (Giannetti et al. 2007; Berger et al. 2009; Porritt et al. 2011; Bouchardet et al. 2014; Magno et al. 2019; Celikel et al. 2024).

Children who did not receive emergency care reported higher prevalence of gingival bleeding and emotional distress. This aligns with findings from Lithuania, where delayed emergency care increased complications and poorer prognosis following TDI (Antipovienė et al. 2021). The psychological impacts observed in the present study, fear, embarrassment, and reluctance to socialize, have also been reported in studies by Lee et al (Lee and Divaris. 2009), Poritt et al (Porritt et al. 2011), and Magno et al (Magno et al. 2019), highlighting the universal emotional burden of TDI. However, concerns have been raised regarding the strength and consistency of existing evidence on the psychological impacts of dental trauma derived from OHRQoL studies. Rodd and Nobel have emphasized the need for future research to identify the paucities, address methodological limitations, and improve the quality of evidence in this area (Rodd and Nobel 2019).

The present study addresses several gaps identified in a prior umbrella review by generating hospital-based evidence, applying a standardized classification system of TDI, examining the influence of trauma severity and delays in reporting, and using a validated OHRQoL instrument appropriate for the enrolled patients. However, there are certain limitations that should be acknowledged. The cross-sectional design limits causal inference, and inclusion of patients with injuries sustained up to 12 months earlier may introduce a degree of recall bias. In addition, differences in the timing of OHRQoL assessment, necessitated by ethical considerations, whereby children with acute severe trauma completed the OIDP questionnaire at a follow-up visit rather than at initial presentation, may also be considered a potential limitation.

## Conclusion

Severe impairment in the OHRQoL, as measured using the OIDP instrument, was observed in approximately 26% of enrolled patients with TDI. The factors such as more severe injuries, soft-tissue involvement, and repeated trauma episodes were associated with poorer OHRQoL outcomes. Although co-existing oral conditions such as dental caries and malocclusion were common in the study population, they did not emerge as significant factors associated with severe OHRQoL impairment. These findings underscore the need to improve awareness of dental trauma prevention and emergency management among parents, caregivers, school-teachers and frontline healthcare providers, as well as to ensure timely access to emergency care. Integrating preventive strategies, early intervention, and psychosocial support into trauma care pathways may substantially reduce the long-term impact of TDI on daily functioning and overall well-being of patients.

## Supplementary Information

Below is the link to the electronic supplementary material.

Supplementary file1 (DOCX 20 KB)

Supplementary file2 (DOCX 23 KB)

## Data Availability

All data supporting the findings of this study are available within the paper and its Supplementary Information.
