# Supplementary material for: Assessment of oral health related quality of life in children and adolescents affected by traumatic dental injuries: a hospital-based study
Source: Eur Arch Paediatr Dent. 2026 Jun 22;27(5):1585–95. doi: 10.1007/s40368-026-01243-6 (PMC13593705; doi:10.1007/s40368-026-01243-6)
Supplement: Supplementary file 2 [file 40368_2026_1243_MOESM2_ESM.docx]

Supplementary File 1: Details of univariate binary logistic regression analysis with dependent variable as total score of OIDP ≥8 (severe) versus the total OIDP score <7 (less severe)

| **var** | **OR** | **95% CI LOR** | **95% CI UOR** | **p value** |
| --- | --- | --- | --- | --- |
| **Age 11-13** | 0.52 | 0.28 | 0.96 | 0.040 |
| **Age 14-16** | 0.70 | 0.32 | 1.48 | 0.367 |
| **Male Gender** | 1.02 | 0.58 | 1.79 | 0.935 |
| **Patients’ education** | 0.67 | 0.38 | 1.19 | 0.170 |
| **Parents’ Education** | 0.98 | 0.73 | 1.32 | 0.902 |
| **Trauma Frequency** | 3.00 | 0.81 | 11.11 | 0.090 |
| **Cause of trauma** | 0.84 | 0.64 | 1.09 | 0.197 |
| **When emergency treatment was provided** | 0.87 | 0.59 | 1.22 | 0.437 |
| **Whether dentist was consulted** | 0.96 | 0.51 | 1.77 | 0.895 |
| **Time elapsed till patient reported for the comprehensive care** | 1.07 | 0.90 | 1.27 | 0.472 |
| **Associated soft tissue injury** | 1.11 | 0.64 | 1.95 | 0.704 |
| **Type of soft tissue injury** | 1.33 | 0.99 | 1.81 | 0.061 |
| **Facial profile** | 0.84 | 0.48 | 1.45 | 0.524 |
| **Lip competency** | 0.87 | 0.46 | 1.59 | 0.651 |
| **Overjet** | 1.01 | 0.76 | 1.31 | 0.963 |
| **Overbite** | 1.12 | 0.82 | 1.53 | 0.464 |
| **Molar relation** | 0.73 | 0.36 | 1.39 | 0.353 |
| **Severity of trauma** | 1.71 | 1.17 | 2.56 | 0.006 |
| **Number of carious teeth** | 1.15 | 0.97 | 1.35 | 0.096 |
| **Presence of sensitivity or tooth ache** | 1.70 | 0.85 | 3.65 | 0.150 |
| **Presence of bleeding gums** | 1.64 | 0.95 | 2.87 | 0.080 |
| **Presence of decayed or broken adult tooth** | 1.81 | 0.58 | 8.01 | 0.359 |
| **Oral ulcer or loose baby tooth** | 1.29 | 0.61 | 2.60 | 0.484 |
| **Problems of tooth colour, shape, size or position** | 2.71 | 1.42 | 5.47 | 0.004 |
| **Difficulties in eating** | 2.39 | 1.75 | 3.34 | 0.000 |
| **Difficulties in speaking** | 6.29 | 3.41 | 12.08 | 0.000 |
| **Difficulties in cleaning mouth** | 3.12 | 2.28 | 4.39 | 0.000 |
| **Difficulties in relaxing (including sleeping)** | 3.18 | 2.33 | 4.48 | 0.000 |
| **Difficulties with feelings (more impatient, irritable, easily upset)** | 2.83 | 2.11 | 3.89 | 0.000 |
| **Difficulties in smiling or laughing** | 3.06 | 2.29 | 4.20 | 0.000 |
| **Difficulties in doing schoolwork** | 4.57 | 2.63 | 8.59 | 0.000 |
| **Difficulty in mixing with friends or other people** | 3.11 | 2.30 | 4.33 | 0.000 |

Supplementary File 2: Details of multivariable binary logistic regression analysis with dependent variable as total score of OIDP ≥8 (severe) versus the total OIDP score <7 (less severe)

| **Variable** | **OR** | **95% CI LOR** | **95% CI UOR** | **p value** |
| --- | --- | --- | --- | --- |
| **Age 11-13** | 0.64 | 0.40 | 1.03 | 0.068 |
| **Age 14-16** | 0.68 | 0.41 | 1.14 | 0.145 |
| **Patients’ education** | 0.68 | 0.42 | 1.12 | 0.127 |
| **Trauma Frequency** | 2.33 | 1.23 | 4.60 | 0.012 |
| **Cause of trauma** | 0.96 | 0.85 | 1.09 | 0.509 |
| **Type of soft tissue injury** | 1.46 | 1.11 | 1.91 | 0.007 |
| **Severity of trauma** | 1.90 | 1.51 | 2.42 | 0.000 |
| **Presence of sensitivity or tooth ache** | 0.68 | 0.46 | 1.00 | 0.049 |
| **Presence of bleeding gums** | 0.81 | 0.49 | 1.34 | 0.421 |
| **Problems of tooth colour, shape, size or position** | 1.70 | 1.22 | 2.37 | 0.002 |
| **Difficulties in eating** | 2.08 | 1.78 | 2.44 | 0.000 |
